# Supplementary material for: Long Noncoding RNA, MicroRNA, Zn Transporter Zip14 (Slc39a14) and Inflammation in Mice
Source: Nutrients. 2022 Dec 1;14(23):5114. doi: 10.3390/nu14235114 (PMC9740689; doi:10.3390/nu14235114)
Supplement: Supplementary file 1 [file nutrients-14-05114-s001.zip › nutrients-2020051-supplementary.pdf]

Table S1. Differential Expression of lncRNAs from RNA-seq.

Symbol	log2FoldChange	LOG(FDR)	padj	*p*-Value	Name
1810065E05Rik	5.877265	8.827E+00	1.49E-09	2.45E-11	RIKEN cDNA 1810065E05 gene
U90926	5.826542	3.954E+00	0.000111	8.02E-06	cDNA sequence U90926
1700057G04Rik	5.640978	1.997E+00	0.010078	0.001843902	RIKEN cDNA 1700057G04 gene
4931440F15Rik	5.579818	6.140E+00	7.24E-07	2.48E-08	RIKEN cDNA 4931440F15 gene
A530053G22Rik	5.316177	2.345E+00	0.004519	0.000689319	RIKEN cDNA A530053G22 gene
2610528A11Rik	4.902529	1.616E+01	6.84E-17	3.06E-19	RIKEN cDNA 2610528A11 gene
Gm9885	3.990235	2.378E+00	0.00419	0.000625727	predicted_gene_9885
RP24-458J4.2	3.6432	1.046E+01	3.48E-11	4.08E-13	RIKEN_cDNA_4732465J04_gene
BC051019	3.596832	4.588E+00	2.58E-05	1.45E-06	cDNA sequence BC051019
Gm16685	3.162146	2.536E+00	0.002908	0.000403567	predicted gene, 16685
9130204K15Rik	3.00091	1.878E+00	0.013237	0.002569172	RIKEN_cDNA_9130204K15_gene
9130024F11Rik	2.992311	6.590E+00	2.57E-07	7.66E-09	RIKEN cDNA 9130024F11 gene
1700020L24Rik	2.768162	2.972E+00	0.001067	0.000120459	RIKEN cDNA 1700020L24 gene
1700086L19Rik	2.720035	2.269E+00	0.005383	0.000858202	RIKEN cDNA 1700086L19 gene
Gm16793	2.681881	6.676E+00	2.11E-07	6.19E-09	predicted gene, 16793
5730507C01Rik	2.471402	1.735E+00	0.018387	0.003861799	RIKEN cDNA 5730507C01 gene
RP24-98O21.6	2.340893	1.455E+00	0.035105	0.008903334	RIKEN_cDNA_2210011K15_gene
Gm13780	2.267305	2.129E+00	0.007427	0.00126404	predicted_gene_13780
Gm16701	2.161842	1.967E+00	0.010777	0.00199579	predicted_gene_16701
H19	2.107413	1.512E+00	0.030793	0.00749242	H19, imprinted maternally expressed transcript
RP23-423G13.11	2.101183	1.877E+00	0.013279	0.00258128	predicted_gene_29367
4930486L24Rik	2.039655	1.412E+00	0.038743	0.010135746	RIKEN cDNA 4930486L24 gene
Gm2061	2.019084	7.358E+00	4.39E-08	1.06E-09	predicted gene 2061
E230025N22Rik	1.914179	1.600E+00	0.025123	0.005760192	Riken cDNA E230025N22 gene
Gm5535	1.892803	1.757E+00	0.0175	0.003620053	predicted gene 5535
9330158H04Rik	1.834922	3.636E+00	0.000231	1.94E-05	RIKEN cDNA 9330158H04 gene
2810032G03Rik	1.800994	1.564E+00	0.027286	0.00641191	RIKEN cDNA 2810032G03 gene
Gm11496	1.735997	1.534E+00	0.029248	0.00702325	predicted_gene_11496
6330403K07Rik	1.708802	4.114E+00	7.70E-05	5.23E-06	RIKEN cDNA 6330403K07 gene
9230105E05Rik	1.683614	1.427E+00	0.037449	0.009699337	RIKEN cDNA 9230105E05 gene
A730056A06Rik	1.661423	1.449E+00	0.035525	0.009059882	RIKEN cDNA A730056A06 gene
A830039N20Rik	1.599297	2.851E+00	0.00141	0.000169049	RIKEN_cDNA_A830039N20_gene
5830444B04Rik	1.571928	2.078E+00	0.008355	0.001466086	RIKEN cDNA 5830444B04 gene
Gm2115	1.551231	2.501E+00	0.003157	0.000444558	predicted_gene_2115
C030013C21Rik	1.548119	1.826E+00	0.014934	0.002986356	RIKEN cDNA C030013C21 gene
9330159F19Rik	1.521146	2.595E+00	0.002544	0.000344546	RIKEN cDNA 9330159F19 gene
5033428I22Rik	1.499011	1.454E+00	0.03513	0.008916288	RIKEN cDNA 5033428I22 gene
1500009L16Rik	1.487643	5.796E+00	1.60E-06	5.98E-08	RIKEN cDNA 1500009L16 gene
9930012K11Rik	1.483285	5.073E+00	8.46E-06	4.03E-07	RIKEN cDNA 9930012K11 gene
B230312C02Rik	1.443431	1.510E+00	0.030938	0.007538528	RIKEN_cDNA_B230312C02_gene
4921536K21Rik	1.392916	2.006E+00	0.009869	0.001793362	RIKEN cDNA 4921536K21 gene
1500011B03Rik	1.388235	3.830E+00	0.000148	1.12E-05	RIKEN cDNA 1500011B03 gene
3300005D01Rik	1.353777	1.439E+00	0.036373	0.00933105	RIKEN cDNA 3300005D01 gene
AI838599	1.247552	1.495E+00	0.032008	0.007876418	expressed_sequence_AI838599

4833412C05Rik	1.226345	1.404E+00	0.039425	0.010395576	RIKEN cDNA 4833412C05 gene
Gm27031	1.178349	1.974E+00	0.010606	0.001957197	predicted_gene_27031
Gm14290	1.142585	2.081E+00	0.008294	0.001452892	predicted_gene_14290
3830403N18Rik	1.136251	1.761E+00	0.017346	0.003575957	RIKEN cDNA 3830403N18 gene
Bvht	1.132362	2.155E+00	0.007004	0.001176691	braveheart long non-coding RNA
2210408F21Rik	1.111202	1.419E+00	0.038125	0.009947128	RIKEN cDNA 2210408F21 gene
Pvt1	1.052021	1.658E+00	0.021957	0.004827978	plasmacytoma_variant_translocation_1
Meg3	1.044219	1.600E+00	0.025132	0.005764594	maternally expressed 3
Gm10687	1.035162	1.580E+00	0.026289	0.006114902	predicted_gene_10687
1700066B19Rik	0.970881	3.808E+00	0.000156	1.20E-05	RIKEN cDNA 1700066B19 gene
B230118H07Rik	0.954014	2.276E+00	0.005294	0.000841093	RIKEN cDNA B230118H07 gene
D430019H16Rik	0.951846	2.003E+00	0.009933	0.001809981	RIKEN cDNA D430019H16 gene
Snhg18	0.940516	2.416E+00	0.00384	0.000563369	small nucleolar RNA host gene 18
RP24-69I6.1	0.933548	3.098E+00	0.000799	8.51E-05	predicted_gene_28933
1700019D03Rik	0.925193	3.287E+00	0.000516	5.01E-05	RIKEN cDNA 1700019D03 gene
5730409E04Rik	0.901814	1.722E+00	0.018971	0.004025518	RIKEN cDNA 5730409E04Rik gene
Gm16938	0.900285	1.310E+00	0.048951	0.013701821	predicted_gene_16938
Mir143hg	0.881084	1.519E+00	0.030266	0.007335229	Mir143_and_Mir145_host_gene_(non-coding_RNA)
Gm17619	0.870118	1.584E+00	0.026075	0.006051931	predicted gene, 17619
1110038B12Rik	0.830935	3.345E+00	0.000452	4.28E-05	RIKEN cDNA 1110038B12 gene
2700097O09Rik	0.741094	2.903E+00	0.001251	0.00014682	RIKEN cDNA 2700097O09 gene
1110059E24Rik	0.687386	3.804E+00	0.000157	1.22E-05	RIKEN cDNA 1110059E24 gene
2200002D01Rik	0.661518	2.684E+00	0.00207	0.000268479	RIKEN cDNA 2200002D01 gene
5330417C22Rik	0.632879	1.655E+00	0.02215	0.004891531	RIKEN cDNA 5330417C22 gene
4930522L14Rik	0.619979	1.958E+00	0.011021	0.002055874	RIKEN cDNA 4930522L14 gene
A330023F24Rik	0.568521	1.497E+00	0.031825	0.007823464	RIKEN cDNA A330023F24 gene
2510009E07Rik	0.544516	1.605E+00	0.024807	0.005670388	RIKEN cDNA 2510009E07 gene
D030056L22Rik	0.500506	1.787E+00	0.016348	0.003325882	RIKEN cDNA D030056L22 gene
9530068E07Rik	0.470946	4.928E+00	1.18E-05	5.93E-07	RIKEN cDNA 9530068E07 gene
1810037I17Rik	0.459347	1.704E+00	0.01976	0.004234637	RIKEN cDNA 1810037I17 gene
6430548M08Rik	0.454934	1.768E+00	0.017073	0.00350676	RIKEN cDNA 6430548M08 gene
C130074G19Rik	0.435969	2.896E+00	0.00127	0.000149401	RIKEN cDNA C130074G19 gene
Tmem250-ps	0.410054	2.085E+00	0.008226	0.001437244	transmembrane protein 250, pseudogene
9530082P21Rik	0.385464	1.334E+00	0.046296	0.012770381	RIKEN cDNA 9530082P21 gene
2810004N23Rik	0.324826	1.353E+00	0.044353	0.012060498	RIKEN cDNA 2810004N23 gene
4930453N24Rik	0.3102	1.510E+00	0.030877	0.007517406	RIKEN cDNA 4930453N24 gene
C330018D20Rik	0.303097	1.397E+00	0.040068	0.010605354	RIKEN cDNA C330018D20 gene
Oip5os1	−0.32283	1.450E+00	0.035459	0.009034018	Opa interacting protein 5, opposite strand 1
0610030E20Rik	−0.34522	2.570E+00	0.002689	0.000368609	RIKEN cDNA 0610030E20 gene
Fendrr	−0.47116	1.426E+00	0.037508	0.00973082	Foxf1 adjacent non-coding developmental regulatory RNA
Gm17491	−0.48885	2.098E+00	0.007983	0.001381206	predicted_gene_17491
AI480526	−0.50073	2.267E+00	0.005413	0.000863832	expressed_sequence_AI480526
BC005537	−0.54327	6.447E+00	3.57E-07	1.10E-08	cDNA sequence BC005537
1810058I24Rik	−0.56704	2.620E+00	0.002397	0.000318824	RIKEN cDNA 1810058I24 gene
D430042O09Rik	−0.56759	5.160E+00	6.92E-06	3.22E-07	RIKEN cDNA D430042O09 gene
2610507B11Rik	−0.59003	3.904E+00	0.000125	9.22E-06	RIKEN cDNA 2610507B11 gene
E230016M11Rik	−0.66373	2.191E+00	0.006445	0.001068455	RIKEN cDNA E230016M11 gene

BC029722	−0.6646	3.013E+00	0.000971	0.00010782	cDNA sequence BC029722
4933431E20Rik	−0.70609	2.963E+00	0.00109	0.000123326	RIKEN cDNA 4933431E20 gene
9430038I01Rik	−0.70671	2.741E+00	0.001817	0.000228464	RIKEN cDNA 9430038I01 gene
9930021J03Rik	−0.7142	2.725E+00	0.001883	0.000238944	RIKEN cDNA 9930021J03 gene
9130019O22Rik	−0.71426	2.622E+00	0.002386	0.00031705	RIKEN cDNA 9130019O22 gene
4833418N02Rik	−0.71697	5.701E+00	1.99E-06	7.83E-08	RIKEN cDNA 4833418N02 gene
Smim31	−0.72374	2.461E+00	0.00346	0.000496865	small integral membrane protein 31
6430550D23Rik	−0.73905	1.353E+00	0.044372	0.0120725	RIKEN cDNA 6430550D23 gene
2310043M15Rik	−0.73956	1.333E+00	0.046442	0.01282953	RIKEN cDNA 2310043M15 gene
Gm26873	−0.75197	1.379E+00	0.04182	0.0111931	predicted_gene_26873
Gm16892	−0.78478	1.404E+00	0.039406	0.010380614	predicted_gene_16892
1700003E16Rik	−0.7965	1.320E+00	0.047833	0.013289911	RIKEN cDNA 1700003E16 gene
9130230L23Rik	−0.82646	1.483E+00	0.032902	0.008164256	RIKEN cDNA 9130230L23 gene
2900005J15Rik	−0.85059	1.455E+00	0.03509	0.008896021	RIKEN cDNA 2900005J15 gene
1700024F13Rik	−0.85137	1.348E+00	0.044838	0.012248791	RIKEN cDNA 1700024F13 gene
BC017158	−0.8676	4.588E+00	2.58E-05	1.45E-06	cDNA sequence BC017158
A930024N18Rik	−0.87852	2.078E+00	0.00835	0.001463948	RIKEN_cDNA_A930024N18_gene
Gm16861	−0.88109	3.004E+00	0.000992	0.000110507	predicted gene, 16861
4931406C07Rik	−0.91752	3.960E+00	0.00011	7.90E-06	RIKEN cDNA 4931406C07 gene
2810408I11Rik	−0.95459	1.428E+00	0.037332	0.009659638	RIKEN cDNA 2810408I11 gene
6430710C18Rik	−0.96252	1.617E+00	0.024156	0.005467011	RIKEN cDNA 6430710C18 gene
F630028O10Rik	−0.98172	1.924E+00	0.011922	0.002274315	RIKEN cDNA F630028O10 gene
2010003K11Rik	−1.04525	4.724E+00	1.89E-05	1.02E-06	RIKEN cDNA 2010003K11 gene
Gm13412	−1.05409	1.467E+00	0.034095	0.008546045	predicted_gene_13412
1600014C10Rik	−1.08127	7.646E+00	2.26E-08	5.11E-10	RIKEN cDNA 1600014C10 gene
Gm16982	−1.09752	1.310E+00	0.048984	0.013717041	predicted gene, 16982
Gm12511	−1.1033	1.828E+00	0.014844	0.002961554	predicted_gene_12511
RP23-401G21.7	−1.1079	1.592E+00	0.025605	0.005914551	predicted_gene_19325
D630044L22Rik	−1.12068	5.330E+00	4.68E-06	2.05E-07	RIKEN cDNA gene D630044L22 gene
1700001L05Rik	−1.12274	3.398E+00	0.0004	3.70E-05	RIKEN_cDNA_1700001L05_gene
4833422C13Rik	−1.1445	2.570E+00	0.002689	0.000368469	RIKEN cDNA 4833422C13 gene
C130050O18Rik	−1.14931	2.514E+00	0.003065	0.000428471	RIKEN cDNA C130050O18 gene
B430306N03Rik	−1.19511	1.564E+00	0.027272	0.006405129	RIKEN cDNA B430306N03 gene
2010106E10Rik	−1.20988	4.975E+00	1.06E-05	5.31E-07	RIKEN cDNA 2010106E10 gene
9930111J21Rik1	−1.23974	1.574E+00	0.026658	0.006218141	RIKEN cDNA 9930111J21 gene 1
9030622O22Rik	−1.2425	3.523E+00	0.0003	2.66E-05	RIKEN_cDNA_9030622O22_gene
A730008H23Rik	−1.25193	3.540E+00	0.000288	2.53E-05	RIKEN cDNA A730008H23 gene
Gm26516	−1.27104	4.103E+00	7.89E-05	5.39E-06	predicted_gene_26516
C030037D09Rik	−1.27841	4.346E+00	4.51E-05	2.80E-06	RIKEN_cDNA_C030037D09_gene
1810012K16Rik	−1.3061	1.919E+00	0.012054	0.00230368	RIKEN cDNA 1810012K16 gene
Gm13710	−1.38658	2.820E+00	0.001512	0.000184333	predicted gene 13710
Gm17529	−1.39201	1.516E+00	0.030473	0.007394459	predicted_gene_17529
1010001N08Rik	−1.45621	1.245E+01	3.52E-13	2.92E-15	RIKEN cDNA 1010001N08 gene
I830077J02Rik	−1.45655	4.807E+00	1.56E-05	8.22E-07	RIKEN cDNA I830077J02 gene
A530064D06Rik	−1.46358	1.360E+00	0.043654	0.011811385	RIKEN cDNA A530064D06 gene
Gm10425	−1.50801	5.963E+00	1.09E-06	3.92E-08	predicted_gene_10425
F730016J06Rik	−1.52701	2.201E+00	0.006295	0.001039165	RIKEN_cDNA_F730016J06_gene

4933405D12Rik	−1.55887	2.240E+00	0.005748	0.000929564	RIKEN cDNA 4933405D12 gene
4732419C18Rik	−1.60577	3.153E+00	0.000703	7.33E-05	RIKEN cDNA 4732419C18 gene
F830208F22Rik	−1.65718	1.383E+00	0.041426	0.011056878	RIKEN cDNA F830208F22 gene
Gm12319	−1.65896	6.770E+00	1.70E-07	4.88E-09	predicted_gene_12319
1810064F22Rik	−1.67901	1.837E+00	0.014541	0.002884267	RIKEN cDNA 1810064F22 gene
1110002E22Rik	−1.68364	2.502E+00	0.003146	0.000442486	RIKEN cDNA 1110002E22 gene
4930552P12Rik	−1.73278	2.878E+00	0.001324	0.000157401	RIKEN cDNA 4930552P12 gene
1500035N22Rik	−1.74437	2.732E+00	0.001854	0.000234389	RIKEN cDNA 1500035N22 gene
4933404O12Rik	−1.95902	9.959E+00	1.10E-10	1.45E-12	RIKEN cDNA 4933404O12 gene
1700071M16Rik	−1.98446	3.953E+00	0.000111	8.06E-06	RIKEN cDNA 1700071M16 gene
Gm11752	−1.99417	1.368E+00	0.042879	0.011554178	predicted_gene_11752
1810055G02Rik	−1.99507	1.040E+01	3.94E-11	4.67E-13	RIKEN cDNA 1810055G02 gene
3300002I08Rik	−1.99826	5.073E+00	8.45E-06	4.02E-07	RIKEN cDNA 3300002I08 gene
1700016C15Rik	−2.06194	5.090E+00	8.13E-06	3.85E-07	RIKEN cDNA 1700016C15 gene
D130043K22Rik	−2.12372	9.059E+00	8.72E-10	1.36E-11	RIKEN cDNA D130043K22 gene
Fam169b	−2.13872	9.780E+00	1.66E-10	2.24E-12	family with sequence similarity 169, member B
2410021H03Rik	−2.2221	2.340E+00	0.004574	0.000700104	RIKEN cDNA 2410021H03 gene
Gm26840	−2.25153	3.670E+00	0.000214	1.76E-05	predicted_gene_26840
Gm13391	−2.3917	1.656E+00	0.022099	0.004873596	predicted_gene_13391
6430503K07Rik	−2.40452	2.113E+00	0.007705	0.001324605	RIKEN cDNA 6430503K07 gene
2210407C18Rik	−2.44841	3.295E+00	0.000507	4.90E-05	RIKEN cDNA 2210407C18 gene
Gm26767	−2.52496	2.718E+01	6.54E-28	6.58E-31	predicted_gene_26767
Gm26608	−2.78573	2.563E+00	0.002736	0.000376126	predicted_gene_26608
9830107B12Rik	−2.81349	2.229E+00	0.005896	0.000960282	RIKEN cDNA 9830107B12 gene
1700042O10Rik	−3.06961	1.952E+00	0.011158	0.002087662	RIKEN cDNA 1700042O10 gene
D830030K20Rik	−3.57006	1.740E+00	0.018205	0.003816145	RIKEN cDNA D830030K20 gene
1700029F12Rik	−3.83038	1.495E+00	0.031973	0.007866158	RIKEN cDNA 1700029F12 gene
4930438A08Rik	−4.09574	1.356E+00	0.044049	0.011960076	RIKEN cDNA 4930438A08 gene
Gm14968	−4.67238	2.378E+00	0.00419	0.000625856	predicted_gene_14968
0610031O16Rik	−5.2208	3.063E+00	0.000866	9.37E-05	RIKEN cDNA 0610031O16 gene

RNA-seq. = RNA-sequencing; padj = adjusted *p*-value.

Table S2. Differential Expression of miRNAs from RNA-seq**.**

Symbol	log2FoldChange	*p*-Value	padj	Name
Dnd1	0.05044	0.89085	0.940178345	microRNA-mediated repression inhibitor 1
Mir194-1	1.04030	0.00157	0.028931722	microRNA 194-1
Mir100hg	0.90823	0.00421	0.019650046	Mir100 Mirlet7a-2 Mir125b-1 cluster host gene
Mir22hg	−0.54418	0.00002	0.000281469	Mir22 host gene (non-protein coding)
Mir99ahg	2.25164	0.00038	0.002749629	Mir99a and Mirlet7c-1 host gene (non-protein coding)
Mirg	0.87612	0.12568	0.256859506	miRNA containing gene
Mirg	0.87612	0.12568	0.256859506	miRNA containing gene
Mir155hg	0.03487	0.96023	0.979784675	Mir155 host gene (non-protein coding)
Mir124a-1hg	1.80649	0.01808	0.060488474	Mir124-1 host gene (non-protein coding)
Mir7027	3.85704	0.00449	0.012749629	microRNA 7027
Mir124-2hg	0.44699	0.88754	0.727496294	Mir124-2 host gene (non-protein coding)

Table S3. Materials.

Reagent or Resource	Source	Identifier
**Antibodies**		
NF-kB	Cell Signaling	Cat#8242; AB_10859369
STAT3	Cell Signaling	Cat#4904; AB_331269
**Chemicals, peptides, and recombinant proteins**		
miRNA All-In-One cDNA Synthesis Kit	abm	Cat#G898
BlasTaq™ 2X qPCR MasterMix	abm	Cat#G891
High-Capacity RNA-to-cDNA™ Kit	Applied Biosystems	Cat#4387406
High-Capacity cDNA Reverse Transcription Kit	Applied Biosystems	Cat#4368813
Protease Inhibitor Cocktail (PIC)	Cell Signaling	Cat#7012
SimpleChIP® Plus Sonication Chromatin IP Kit	Cell Signaling	Cat#56383
SimpleChIP® Universal qPCR Master Mix	Cell Signaling	Cat#88989
Matrigel® Matrix	Corning	Cat#356231
Glycine	Fisher	Cat#BP381
Ultrapure DNase/RNase free Distilled Water	Fisher	Cat#BP561
All-in-One™ miRNA qRT-PCR Detection Kit 2.0	GeneCopoeia	Cat#QP016
All-in-One™ miRNA First-Strand cDNA Synthesis Kit	GeneCopoeia	Cat#QP018
DMEM/F-12, HEPES	Gibco	Cat#11330032
Gentamicin	Gibco	Cat#15750060
TURBO DNA-free™ Kit	Invitrogen	Cat#AM1907
PureLink™ RNA Mini Kit	Invitrogen	Cat#12183018A
EXPRESS SYBR™ GreenER™ qPCR Supermix, with premixed ROX	Invitrogen	Cat#11794200
EXPRESS One-Step Superscript™ qRT-PCR Kit, with premixed ROX	Invitrogen	Cat#11791200
TRI Reagent®	Molecular Research Center	Cat#TR118
RNeasy Mini Kit	Qiagen	Cat#74104
miRNeasy Tissue/Cells Advanced Mini Kit	Qiagen	Cat#217604
RNeasy Plus Mini Kit	Qiagen	Cat#74134
Cultrex Organoid Harvesting Solution	R&D Systems	Cat#370010001
Gentle Cell Dissociation Reagent	StemCell	Cat#1000485
IntestiCult™ Organoid Growth Medium (Mouse)	StemCell	Cat#06005
Zinc Sulphate	Thermo Scientific	Cat#205982500
Glycerol	Thermo Scientific	Cat#17904
Halt™ Protease and Phosphatase Inhibitor Cocktail	ThermoScientific	Cat#78442
RNase A	ThermoScientific	Cat#EN0531
Nitric Acid	VWR	Cat#936801
ChIP DNA Clean & Concentrator™	Zymo Research Corporation	Cat#D5205
DNase I	Zymo Research Corporation	Cat#E1010
**Deposited data**		
Gene Expression Omnibus	GSE210160	
Protocol number	202007015	
**Experimental models: Organisms/strains**		
C57BL/6 mice (WT)	JAX	000664
Zip14$^{-/-}$ knockout mice (WB-KO)	In house	
Zip14$^{flox/+}$ mice	MMRRC:UC Davis	custom
Zip14$^{flox/flox}$ mice (F/F)	In house	
Villin-Cre (B6.Cg-Tg(Vil1-cre)997Gum/J)	JAX	004586
Villin-Cre (VC- Zip14$^{+/+}$) mice	In house	
Zip14$^{\Delta IEC}$ mice (ΔIEC)	In house	

**Oligonucleotides**		
lnc-U90926 For: TTCTCTGACTGGGGGTCCTC<br>lnc-U90926 Rev: AGCTGGAAGCATGATCCGAC	10.1038/ijo.2016.189	N/A
lnc-H19-F: AGAGGACAGAAGGGCAGTCA<br>lnc-H19-R: TGGGTGGACAATTAGGTGGT	10.1371/journal.pone.0056611	N/A
Lnc-U90926 For: TTCTCTGACTGGGGGTCCTC<br>Lnc-U90926 For: AGCTGGAAGCATGATCCGAC	10.1038/ijo.2016.189	N/A
mS18 F: CGGAAAATAGCCTTCGCCATCAC<br>mS18 R: ATCACTCGCTCCACCTCATCCT	OriGene	CAT#: MP212910
mGapdh F: CATCACTGCCACCCAGAAGACTG<br>mGapdh R: ATGCCAGTGAGCTTCCCGTTCAG	OriGene	CAT#: MP205604
mMeg3 F: TGAAGAACTGCGGATGGAAG<br>mMeg3 R: CACGTAGGCATCCAGGTGAT	10.3892/ol.2019.10969	N/A
mPvt1-F: CTCAGCAGATGTCACACAGACG<br>mPvt1-R: AGGGTCAGTATCATGGCTGGAT	this paper	N/A
mBvht-F: AGGCTGAAGCAAAGCAAGTT<br>mBvht-R: GGCTTACAGTCGGCTTTTCCT	this paper	N/A<br>N/A
mNEAT1-F: CTTGCCACACCTTGTCTTGC<br>mNEAT1-R: TAGCTGGTGCATCCTGTGTG	this paper	N/A
chip_stat3-nfkb/u90926 F: GATCCTTGCAAGAAGCACCCAG<br>chip_stat3-nfkb/u90926 R: GACTCTCTTTGGGTTGTTTGGTTT<br>chip_stat3-nfkb/u90926 For: GACTGGAGCGAGCGGTATC<br>chip_stat3-nfkb/u90926 Rev: CCGTTGGTGAATCTTGGAT-TTCTTT	this paper	N/A
chip_stat3/Meg3 F: CACGAGTGTGCGGTCATTTC<br>chip_stat3/ Meg3 R: AGACACTAGGCTACCCCCTT<br>chip_stat3/Meg3 For: GGATGTCCGGGAGACTTTGA<br>chip_stat3/ Meg3 Rev: CTAGGCTACCCCCTTTGACAC	this paper	N/A
chip_nfkb/Meg3 F: GAGAGGAGGGGTGAATTGGC<br>chip_nfkb/ Meg3 R: GAGTCCAAGTGTCCTCCGTG<br>chip_nfkb/Meg3 For: GGAAAGAGAACTGGGAGAGCC<br>chip_nfkb/ Meg3 Rev: TGTGGCAAGAGTCCAAGTGT	this paper	N/A
chip_stat3/H19 F: TGCCCTGATTTGTGGATGCT<br>chip_stat3/ H19 R: CGTGCGTGCGTATACCATTG<br>chip_stat3/H19 For: AAACGTGACGACTCACCCAG<br>chip_stat3/ H19 Rev: TTATCTGGCCGCAGTGGTTT<br>chip_stat3/H19.1 For: ATACTGTGCCTCCGGTTGAC<br>chip_stat3/ H19.1 Rev: CTCACCGTCCCTTTTCTGGG	this paper	N/A
mSTAT3-H19 For: GACTGGTCAGCCCTTGAGTC<br>mSTAT3-H19 Rev: AGCATCCACAAATCAGGGCA<br>mSTAT3-H19_F: GAGCTCCGGTCACTTCCATC<br>mSTAT3-H19_R: CCACAGAGTCAGCATCCACA	this paper	Same primers were used for H3k4me3, H3k27me3
miR-675-3P	abm	Cat#MPM
miR-675-5P	abm	Cat#MPM
miR-7027-3P	abm	Cat#MPM
miR-7027-5P	abm	Cat#MPM
**Software and algorithms**		
HiSat2	10.1038/nmeth.3317	
Trimmomatic	10.1093/bioinformatics/btu170	
FastQC	BaseSpace Labs	
Prism 9.0	Dotmatics	
DESeq2	10.1186/s13059-014-0550-8	
R software	https://www.r-project.org/<br>(accessed on 23 October 2022)	

Illumina NovaSeq 6000	Illumina	
Illumina MiSeq	Illumina	
Aperio ImageScope	Leica Biosystems	
Primer Blast tool	NCBI	
ImageJ	NCBI	
Primer Blast tool	NCBI	
ImageJ	NCBI	
Scaffold 5	Proteome Software	
SAMtools	SAMtools software	
SILVA_132	Silva Databases	
Proteome discoverer™ Software	Thermo Scientific	
**Other**		
Orbital shaker		
Centrifuge		
BioAnalyzer	Agilent	
Microwave Plasma-Atomic Emission Spectophotometer	Agilent Technologies	MPAES-4210
Bioruptor sonicator	Diagenode	
Rodent Diet	Harlan	7012
Leica DM4000B digital microscope	Leica Biosystems	
SpectraMax M5	Molecular Devices	
Bullet Blender	Next Advance	
Zirconium Oxide Beads	Next Advance	ZrOB10-RNA
Stainless Steel Beads	Next Advance	SSB14B
FluorChem E	Protein Simple	
Z Gel micro tubes	Sarstedt	41.1500.005
Evos fl	ThermoFisher	
Nanodrop™ one	ThermoFisher	
QuantStudio3 qPCR	ThermoFisher	
Orbitrap Fushion Tribrid Mass Spectrometer System	ThermoFisher	
Ultra-performance LC system	ThermoFisher	
PepMap RSLC analytic column	ThermoFisher	
Incubator series 3 (cell culture)	ThermoFisher	
Isotemp 205	ThermoFisher	

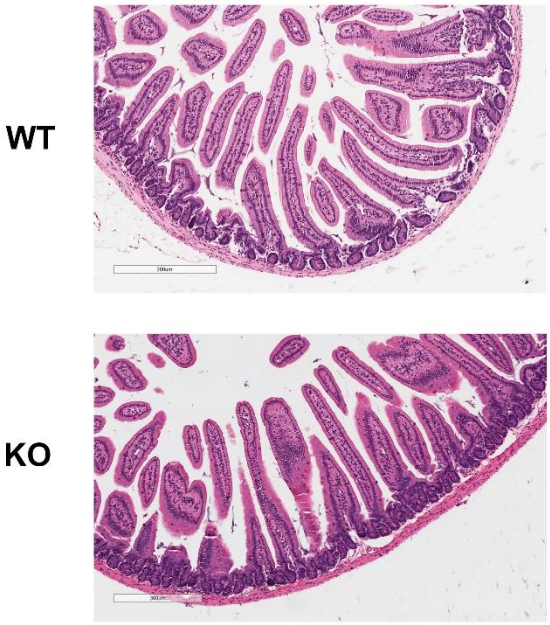

Figure S1. Histopathology of small intestine from whole body Zip14 knockout and WT mice. H&E-stained tissue sections of duodenum. KO = knockout mice; WT = wild-type mice; H&E = haemotoxylin and eosin.
